# Feasibility of utilizing the SD BIOLINE Onchocerciasis IgG4 rapid test in onchocerciasis surveillance in Senegal

**DOI:** 10.1371/journal.pntd.0005884

**Published:** 2017-10-03

**Authors:** Yakou Dieye, Helen L Storey, Kelsey L. Barrett, Emily Gerth-Guyette, Laura Di Giorgio, Allison Golden, Dunia Faulx, Michael Kalnoky, Marie Khemesse Ngom Ndiaye, Ngayo Sy, Malang Mané, Babacar Faye, Mamadou Sarr, Elhadji Mamadou Dioukhane, Roger B. Peck, Philippe Guinot, Tala de los Santos

**Affiliations:** 1 PATH, Seattle, Washington, United States of America; 2 Senegal Ministry of Health and Social Action, Dakar, Senegal; 3 University of Cheikh Anta Diop, Dakar, Senegal; 4 Medical Region of Kédougou, Kedougou, Senegal; Swiss Tropical and Public Health Institute, SWITZERLAND

## Abstract

As effective onchocerciasis control efforts in Africa transition to elimination efforts, different diagnostic tools are required to support country programs. Senegal, with its long standing, successful control program, is transitioning to using the SD BIOLINE Onchocerciasis IgG4 (Ov16) rapid test over traditional skin snip microscopy. The aim of this study is to demonstrate the feasibility of integrating the Ov16 rapid test into onchocerciasis surveillance activities in Senegal, based on the following attributes of acceptability, usability, and cost. A cross-sectional study was conducted in 13 villages in southeastern Senegal in May 2016. Individuals 5 years and older were invited to participate in a demographic questionnaire, an Ov16 rapid test, a skin snip biopsy, and an acceptability interview. Rapid test technicians were interviewed and a costing analysis was conducted. Of 1,173 participants, 1,169 (99.7%) agreed to the rapid test while 383 (32.7%) agreed to skin snip microscopy. The sero-positivity rate of the rapid test among those tested was 2.6% with zero positives 10 years and younger. None of the 383 skin snips were positive for Ov microfilaria. Community members appreciated that the rapid test was performed quickly, was not painful, and provided reliable results. The total costs for this surveillance activity was $22,272.83, with a cost per test conducted at $3.14 for rapid test, $7.58 for skin snip microscopy, and $13.43 for shared costs. If no participants had refused skin snip microscopy, the total cost per method with shared costs would have been around $16 per person tested. In this area with low onchocerciasis sero-positivity, there was high acceptability and perceived value of the rapid test by community members and technicians. This study provides evidence of the feasibility of implementing the Ov16 rapid test in Senegal and may be informative to other country programs transitioning to Ov16 serologic tools.

## Author summary

As onchocerciasis control programs succeed and transition to elimination efforts, different diagnostic tools are needed. The goal of this study was to determine if integrating the Ov16 rapid test is feasible based on acceptability, usability, and cost. A study was conducted in 13 villages in southeastern Senegal in May 2016. Community members were invited to participate in a demographic questionnaire, a rapid test, a skin snip biopsy, and an acceptability interview. Technicians were also interviewed and a costing analysis was conducted. Out of 1,173 participants, 1,169 (99.7%) agreed to the rapid test while 383 (32.7%) agreed to skin snip microscopy. The rapid test result was reactive in 2.6% of those tested, while none of the skin snips were positive. Community members thought the rapid test was performed quickly, was not painful, and provided reliable results. If no one had refused skin snip microscopy, the total cost would have been around $16 per person tested for either method. In this area with little if any remaining onchocerciasis, there was high acceptability and perceived value of the rapid test. This study suggests that implementing the Ov16 rapid test in Senegal is feasible and these findings may be informative to other country programs.

## Introduction

Onchocerciasis, commonly known as river blindness, is caused by the filarial parasite *O*. *volvulus* (Ov) that affects an estimated 37 million people, with an estimated 187 million living in areas at risk of infection, primarily in Africa.[1,2] An estimated 1.1 million disability-adjusted life years (DALYs) were lost in 2015 due to onchocerciasis, as it can lead to severe and disfiguring skin disease, visual impairment, and eventually blindness.[3] Onchocerciasis especially affects poor rural communities and the risk of infection is substantially higher among socioeconomically disadvantaged groups.[4] In Africa, efforts to date have focused primarily on disease control through mass drug administration (MDA) with ivermectin, an antiparasitic drug donated by Merck.[5,6]. Recent evidence from Sudan, Senegal, Mali and Uganda suggests elimination is possible in Africa as it is in the Americas.[6–11]

In response to this success, the global strategy has shifted from disease control to disease elimination.[6,12] The 2016 World Health Organization (WHO) guidelines on stopping MDA and verifying elimination describe three phases of onchocerciasis elimination programs that require different diagnostic tools: transmission suppression, transmission interruption, and transmission elimination.[13] The standard method is direct observation of the Ov microfilaria in a skin snip biopsy using microscopy. Skin snip microscopy is highly specific and able to detect active infections, but has diminished sensitivity in low-prevalence settings. As the prevalence of onchocerciasis in endemic communities decreases, more sensitive diagnostic tests are needed.[14]

Ov16 serology, used to detect IgG antibodies to the Ov16 antigen in a sentinel population of children under ten years, is now recommended to determine if interruption of transmission of Ov has occurred.[13] Laboratory-based Ov16 ELISA (enzyme-linked immunosorbent assay) is one method to measure these markers, though it requires collecting samples in the field to transport to a laboratory setting for analysis. Currently, there is no standardized commercially available Ov16 ELISA so variations in protocols and procedures exist across labs.[15,16] In 2014, a field deployable, rapid diagnostic tool that could be more easily integrated into current onchocerciases surveillance programs in endemic countries was developed and made commercially available (SD BIOLINE Onchocerciasis IgG4 rapid test, referred to here as Ov16 rapid test).[17,18] Performance of the Ov16 rapid test continues to be evaluated in the field and current global research priorities focus on operational and implementation research to demonstrate utility and increase access of the Ov16 rapid test, particularly in low prevalence settings which have undergone multiple rounds of MDA.[14,19]

In Senegal, MDA and surveillance has been ongoing since 1988 and has resulted in the successful control of onchocerciasis.[7–9] Additionally, MDA for onchocerciasis and lymphatic filariasis (LF) are now integrated. After over 25 years of control efforts, program managers require more clarity around whether transmission has been interrupted. Though skin snip microscopy has been used to this point, it is a painful and invasive procedure that may result in decreased participation in surveillance activities in communities where decades of testing have occurred. Implementation research on the Ov16 rapid test in Senegal was desired to support the program transition to elimination. In 2015, a workshop was held with representatives from the Senegal Ministry of Health and Social Action (MoH) to discuss the current process for using skin snip microscopy and evaluate the potential process if they were to use the rapid test, to streamline introduction of the new test. Acceptability, usability and costing data was also needed to inform decisions on use of the tests in surveillance activities.

The aim of this study is to demonstrate the feasibility of integrating the Ov16 rapid test into onchocerciasis surveillance activities in Senegal, based on the attributes of acceptability, usability, and cost. Quantitative and qualitative methods are used to evaluate the following outcomes: 1) the diagnostic results of the Ov16 rapid test compared to skin snip microscopy; 2) an assessment of the acceptability and usability of the rapid test among community members and health workers; and 3) an estimation of the economic costs to conduct a surveillance activity by diagnostic method from the government’s point of view. A recently developed comprehensive quality assurance (QA) program was also piloted to support proper use of the rapid tests. This implementation research is intended to build evidence to support the introduction of the Ov16 rapid test in Senegal, as well as to inform other settings that may be at a comparable phase of elimination programming.

## Methods

### Study design and population

A cross-sectional study using qualitative and quantitative methods was conducted to assess the feasibility of integrating the Ov16 rapid test into ongoing surveillance activities. The study was conducted along with the Senegal MoH, which currently utilizes skin snip microscopy for onchocerciasis diagnosis. The study was performed in 13 villages in the Kédougou and Saraya districts of southeastern Senegal in May 2016. Villages were representative of the region endemic for onchocerciasis in Senegal, and are co-endemic for LF. These communities started MDA with ivermectin (IV) in 1988. Albendazole was added to the MDA in 2015 and was last administered in these villages in March 2015. Individuals 5 years and older were invited to participate in any or all components of the study, including a demographic and health history questionnaire, the Ov16 rapid test, two skin snip biopsies for microscopy, and an exit interview. Community sensitization was conducted in each village 2–3 days prior to the surveillance activity.

### Ethics statement

This study was approved by the PATH Research Ethics Committee and the Senegal National Ethics Committee for Health Research. Informed consent or assent was obtained from all participants. All participants 18 years and older provided written informed consent, and all participants under 18 years provided assent in addition to their parent or guardian providing written informed consent.

### Diagnostic testing

Prior to study start, a comprehensive quality assurance program was introduced and a training on proper use of the Ov16 rapid test was conducted. The QA program includes training resources such as videos and PowerPoint slides, as well as a quality assurance panel to verify a quality product was received, and daily quality controls to ensure proper functioning of the test throughout data collection. For more information: http://sites.path.org/dx/ntd/training-and-qaqc-materials/. The Ov16 rapid test was performed per the product instructions, which involves transferring 10 μL of finger stick capillary blood to the cassette using a disposable capillary tube that is included with the test. After buffer is added to the cassette, the test runs for 20 minutes and then results are recorded. All rapid test results were read a second time the next day as a research activity to compare 20 minute and overnight results. Skin snip microscopy was performed by taking two skin snips from the iliac crests with a sterile 2 mm corneoscleral punch biopsy tool. The skin snips were incubated in distilled water for 30 minutes, then examined under a light microscope to detect the presence of Ov microfilaria. Skin snips that were negative at 30 minutes were incubated in saline for 24 hours and examined again by microscope to confirm the negative result.

### Diagnostic results analysis

A demographic and health history questionnaire was completed for all participants. Data were entered directly into a mobile-phone based data collection application developed using the Open Data Kit (ODK) 2.0 software, and captured village-specific GPS coordinates as well.[20] Rapid test and skin snip microscopy data was also recorded in the data collection application, including any refusals to perform a test and reasons for that refusal. As rapid test results were not available for at least 20 minutes, individuals who refused the skin snip or rapid test did so prior to knowledge of their test results. Characteristics of participants were reported as proportions for dichotomous variables, and median (interquartile range) or mean (standard deviation) for continuous variables. Sero-positivity of Ov16 rapid test was evaluated using equally distributed age categories. Age was also evaluated as a confounder for continuous and dichotomous variables using linear and logistic regression, respectively. Logistic regression was used to determine associations between exposure characteristics and rapid test result, adjusted for age (Table 1). Participation rates for the two diagnostic methods were compared by McNemar test. Questionnaire data was analyzed using StataSE version 13.1.

**Table 1:** Rapid test results by exposure characteristics among those who participated in rapid test (n = 1,169).

Exposure characteristics	Rapid test positive, ≤20 years, (n = 3/775, 0.4%)		Rapid test positive, >20 years, (n = 27/394, 6.9%)		Rapid test negative (n = 1,139/ 1,169, 97.4%)		Odds of rapid test positive ^1^, 95% Confidence Interval (n = 1,169)
Length of time lived in village							
More than 1 year ^2^	100.0%		100.0%		99.1%		1
Born in village (n = 1,159) ^2^	100.0%		37.0%		80.8%		0.55 (0.24–1.25)
If not born in village, years lived in village (n = 226, mean, SD) ^2^			27.9	(11.4)	18.0	(13.9)	1.03 (0.98–1.08)
Lived outside of village in last 10 years (n = 1,158) ^2^	0.0%		11.1%		7.9%		1.30 (0.38–4.51)
Stream access							
Stream near the village	100.0%		100.0%		99.2%		1
Go to the stream (n = 1,160) ^2^	100.0%		96.3%		93.0%		7.04 (0.86–57.53)
Frequency of going to stream (n = 1,088) ^2^							
Everyday	66.7%		48.2%		58.3%		0.70 (0.21–2.29)
1–3 times a week	33.3%		23.1%		30.5%		0.75 (0.26–2.13)
Less than 1 time per week	0.0%		25.9%		6.3%		Reference
Ivermectin (IV) use							
IV distribute in village in past (n = 1,074)	100.0%		88.9%		82.2%		1.25 (0.28–5.50)
Year IV last distributed (n = 924, mean, SD) ^2^	2012 (4.0)		2009 (3.3)		2012 (3.3)		**0.83 (0.74–0.93)**
Ever taken IV (n = 945) ^2^	66.7%		85.2%		59.5%		5.91 (0.76–45.13)
IV distributed this/last year (n = 688) ^2^	66.7%		7.4%		14.8%		1.07 (0.34–3.38)
Took IV this/last year (n = 167)	66.7%		7.4%		14.1%		1
Presence of oncho symptoms							
Itchy skin (n = 1,164) ^2^	0.0%		22.2%		11.7%		1.09 (0.41–2.86)
Nodules under skin (n = 1,167)	0.0%		0.0%		0.1%		1
Other skin changes (n = 1,168) ^2^	0.0%		0.0%		0.7%		1
Changes in vision (n = 1,165) ^2^	0.0%		51.9%		13.4%		1.69 (0.73–3.93)

^1^ Adjusted for age

^**2**^ Exposure characteristic associated with age (p<0.05)

### Acceptability and usability analysis

Targeted members of the surveillance team and community members participating in surveillance activities were interviewed to provide feedback on the acceptability and usability of the rapid test. All rapid test technicians, were asked questions regarding their experience in using the tests. Community members were sampled purposively based on the diagnostic testing they participated in as well as their willingness to participate in an exit interview. A semi-structured interview guide was used to gather data on the user experience, how the test was received by participants, and how the test compared to experiences with skin snip microscopy. Interviews with community members and technicians were recorded as audio files, then transcribed and translated from local languages into French and then into English. Interview data were coded using content analysis based on key themes from the semi-structured interviews.[21] Refinements were made to the codebook in an iterative fashion during the analysis process and reviewed by two researchers who reached consensus on the findings. Interview data was analyzed using NVivo version 10.

### Costing analysis

A costing analysis was conducted to assess the costs related to the implementation of the onchocerciasis surveillance activity in Senegal by diagnostic test. The study focuses on the economic costs from the government’s perspective, therefore valuing volunteers’ time. Data was mainly gathered from secondary sources such as financial reports, consolidated budgets, and other secondary sources of financial information from PATH and from the Senegalese onchocerciasis surveillance team. A simple structured questionnaire was also used to identify resources used during surveillance activities that had been purchased in previous years. Where needed, costs were calculated using the ingredient approach, multiplying the input prices by the number of inputs used.[22,23] Key input prices for this analysis are: Ov16 rapid test ($1.20), skin snip tool ($225), and microscope ($2,490). Costs were captured for all activities conducted during the surveillance activity, including training, field work, and data reporting. Field work cost categories were further split into labor, supplies, devices and instruments, transport and lodging, and data reporting. Drugs costs were zero since none of the study participants tested positive by skin snip microscopy, which would indicate treatment according to standard of care. The identified resources used were then allocated to the rapid test, the skin snip microscopy, or to shared costs, which were costs that were incurred independent of the type of test used, such as data entry and analysis, or transport cost to the villages.

Total costs were first calculated by cost category and then aggregated across categories. The costs per test performed were calculated by dividing the total costs by the number of participants evaluated with each test. We also estimated the costs assuming the same population size (n = 1,169) for both tests by proportionally scaling up the variable costs for skin snip microscopy, while keeping the fixed costs constant. This was done because of the difference in the number of participants tested with the rapid test (n = 1,169) compared to skin snip microscopy (n = 383) and the presence of high fixed costs of devices and instruments, allowing a comparison of the costs without the volume effect. All cost estimates are presented in $US using an exchange rate of 591.45 XOF per $US (World Bank Development Indicators, World Bank). Further details on the cost analysis is available **as supporting information** (S1 File)

## Results

### Diagnostic results of the rapid test compared to skin snip microscopy

Of the 1,173 participants who agreed to participate in the study, the median age was 12 years and ranged from 5 to 92 years. The most common professions were farmer, student and housewife. Participation rates for the two diagnostic tests differed with a total of 1,169 participants (99.7%) agreeing to be tested by the rapid test and 383 participants (32.7%) agreeing to be tested by skin snip microscopy (p<0.0001). (Table 2)

**Table 2:** Characteristics of 1,173 included participants by gender.

Characteristic	Percent or median (IQR)			
	Male (n = 598)		Female (n = 575)	
Age	12	(23)	12	(21)
Primary profession				
Farmer	27.6%		13.7%	
Merchant	0.3%		0.4%	
Fisherman	0.5%		0	
Student	61.5%		51.3%	
Housewife	0		27.0%	
Other	10.0%		7.7%	
District				
Kedougou	58.9%		58.3%	
Saraya	41.1%		41.7%	
River basin				
Gambia	58.9%		58.3%	
Faleme	37.5%		37.4%	
Koilakabe	3.7%		4.4%	
Participated in rapid test	99.8%		99.5%	
Participated in skin snip microscopy	32.9%		32.4%	

**Fig 1:**
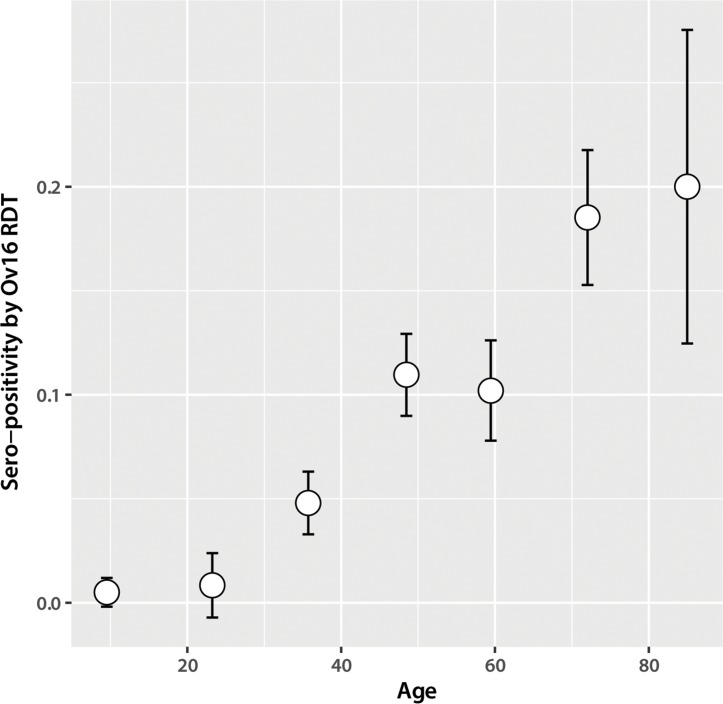
Sero-positivity of Ov16 rapid test by the following age categories: 5–17, 18–30, 31–42, 43–55, 56–67, 68–80, and 81–92 (n = 1,169).

The sero-positivity rate of the rapid test among those who performed the test was 2.6% (30/1,169) among all ages (age range of positives, 11–81), 0.4% (3/775) among 20 years and under, and 6.9% (27/394) among those over 20 years. The 20 year breakpoint was evaluated based on the distribution of the data. (Fig 1) All results were either positive (sero-positive) or negative, as no invalid results were detected. There were zero positive skin snip results. Age was associated with the rapid test result (p<0.001) and 13/17 exposure characteristics, though it was not associated with refusal to participate in either diagnostic test. In an age adjusted analysis, odds of having a rapid test positive result decreased the more recently IV was distributed in their village (0.83, 95% CI: 0.74–0.93). The 3 participants under 20 years who had a positive rapid test result had all lived in their villages their whole lives, frequently went to the stream near their village, and did not report experiencing any of the onchocerciasis symptoms that were included in the questionnaire. Two of the 3 individuals reported having taken IV in the last year. (Table 1)

### Acceptability and usability of the rapid test among community members

**Fig 2:**
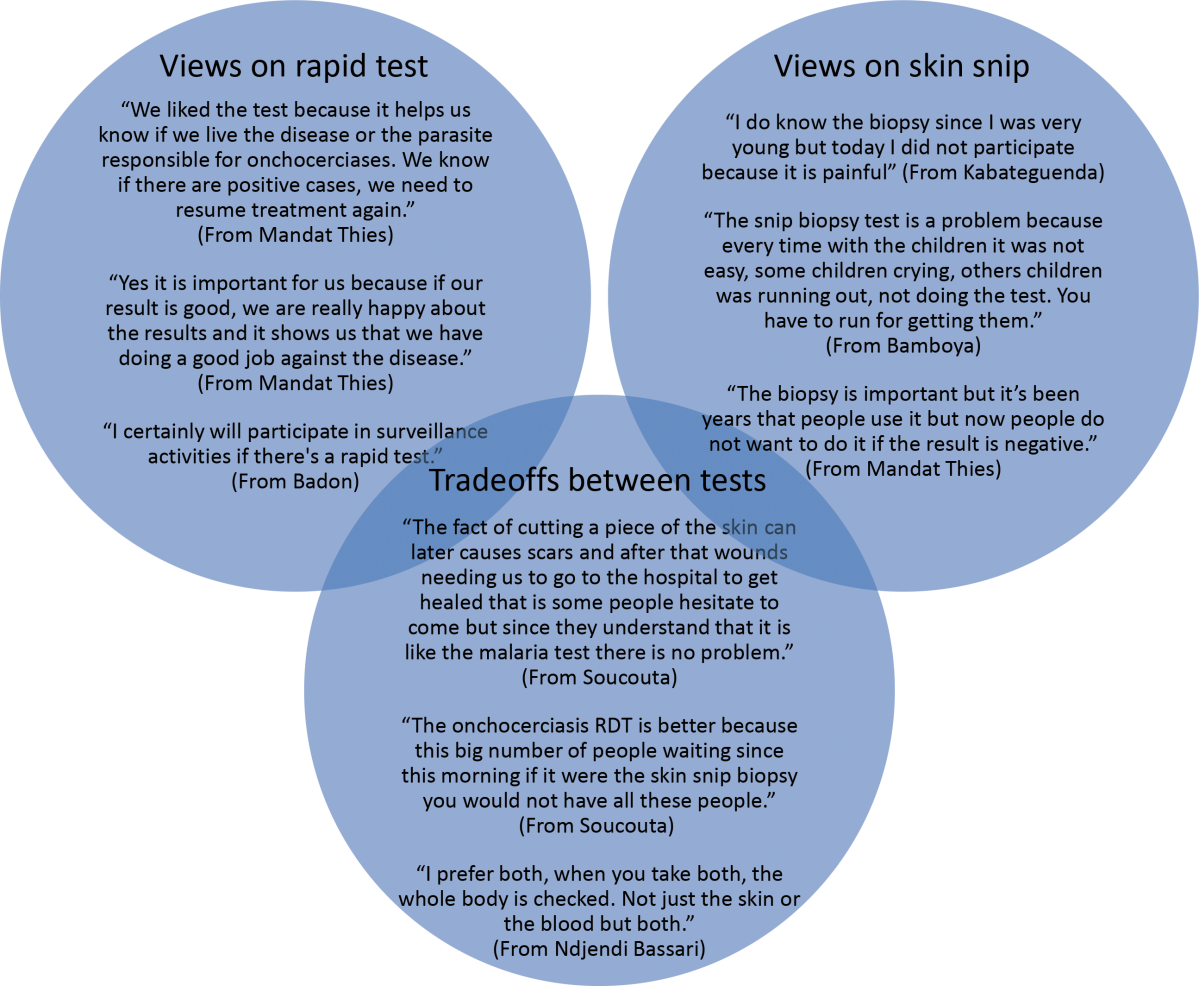
Representative quotes illustrating community member views on the different diagnostic methods.

Interviews were conducted with 4–5 community members from all 13 villages (n = 61). Over 90% of participants (57/61) reported that they valued or appreciated the rapid test. Community members liked that the test was performed quickly and was not painful, and they perceived it to provide reliable results. Community members noted that the test brought health knowledge to the community, enabled them to access follow-on care if needed, and could effectively be used to test children. Most participants (55/61) reported that they had no concerns about the rapid test while 10 percent of participants (6/61) disliked the finger stick component of the rapid test procedure. Moreover, many participants indicated that they would be more likely to participate in future surveillance activities if the rapid test was used and suggested that its use would spur broader participation within the community. The more common reasons for refusing the skin snip biopsy were that they “did not like the idea” and “thought it would be too painful”. Some community members discussed historical experiences with the biopsy procedure like it being painful and suggested that now, they are less willing to undergo the biopsy if the results are consistently negative. With regards to preferences for either diagnostic tool, 50% of respondents preferred the rapid test to skin snip microscopy, 39% expressed liking both tests, and 10% preferred the skin snip microscopy. The primary reasons were that the rapid test was less painful, quicker, and could provide individual test results. Some respondents, particularly those who were sensitized to the differences between the principles of each test, wanted to continue to use both tests or preferred the skin snip microscopy because it could provide a confirmation of infection. (Fig 2)

Community members also noted that the role of the surveillance team and the information they provided influenced their experience with the test. Nearly all of the participants indicated that the surveillance team was skilled and they trusted their abilities. For some, the skills of the surveillance team translated to credibility of the test itself and trust in the test result. The information that the surveillance team provided to community members regarding the test procedure and the test results varied among participants. Some felt like the test and their result were well-explained while others reported not learning their test results. Community members expressed a strong preference for understanding the test procedure and purpose as well as their results. Participants overwhelmingly preferred to receive their individual results though a minority of respondents also wanted to receive sero-prevalence results to understand the health status of the entire community. Community members also noted appreciation for the health services being made available in their village.

### Acceptability and usability of the rapid test among the surveillance team

Interviews were conducted with all rapid test technicians (n = 7). All technicians commented that they liked using the rapid test and most preferred it to skin snip microscopy as they perceived it to be more reliable and quicker to complete. They noted that it was less painful for participants, and thus made their jobs easier as community members were more willing to participate in surveillance. When prompted, one technician reported that the disposable capillary pipette was difficult to use; no other challenges were reported. Most of the rapid test technicians trusted the results of the test, in part due to emphasis on the quality assurance program throughout the study. However, these technicians noted some of the limitations of an antibody test and one technician stated a preference for skin snip microscopy to confirm infections. All technicians indicated that they would be willing to use the rapid test in future surveillance activities.

### Costs per rapid test and per skin snip microscopy

**Fig 3:**
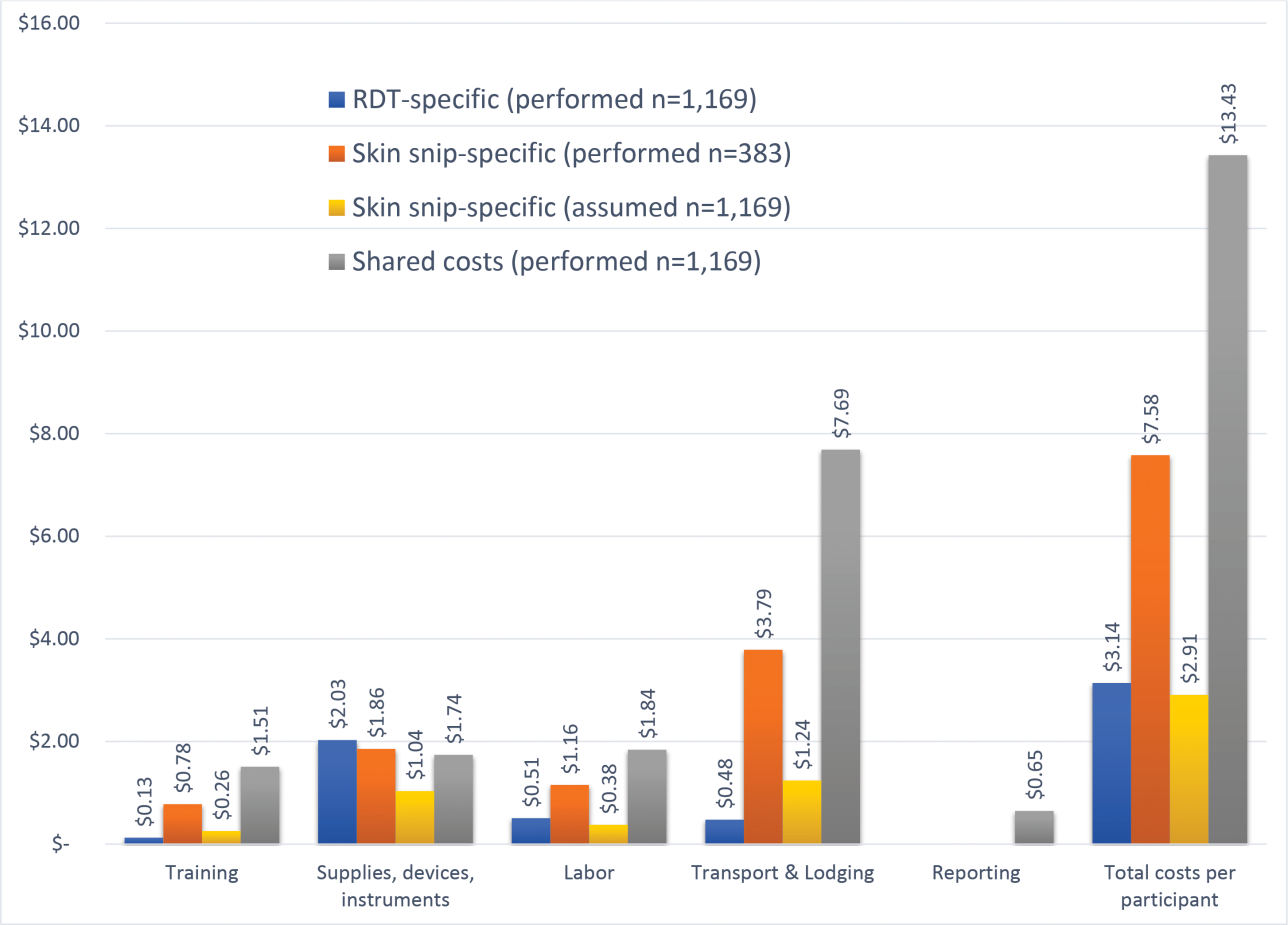
Onchocerciasis surveillance costs per test and by cost category.

The total costs for the onchocerciasis surveillance activities in the 13 villages was estimated at $22,272.83. Costs were allocated to rapid test, skin snip microscopy, or shared costs. Shared costs were those incurred independent of the diagnostic test used and accounted for 70% of the total study costs ($ 15,697.48). Total test-specific costs were $3,671.76 for rapid test and $2,903.59 for skin snip microscopy, though the number of tests performed with each method differed (1169 and 383, respectively). Most of the total study costs (87%) were related to the field work activities, while training costs accounted for 10% of the total costs and data entry and reporting was 3% of the total costs. Of the field work costs, the main cost driver was transport costs (57%), followed by supplies, instruments, and devices (27%). In this surveillance activity, the total cost per test performed was $3.14 for rapid test, $7.58 for skin snip microscopy, and $13.43 for shared cost, giving a total cost per person tested of $16.57 for the rapid test, $21.01 for the skin snip microscopy, and $24.15 if both diagnostic tests were performed on the same participant. If no participants had refused the skin snip microscopy, so the same number of participants were tested with both diagnostic tests, the cost per person tested by skin snip microscopy would have decreased to $2.91. Adding in shared costs, the total cost per participant in the surveillance activity would have been $16.57 for the rapid test and $16.34 for skin snip microscopy, a difference of $ 0.23 per method. (Fig 3)

## Discussion

This study demonstrates that the inclusion of the rapid test in surveillance activities is feasible based on acceptability, usability, and costs. The sero-positivity rate of the rapid test among those who performed the test was 2.6% among all ages with no positives detected under 10 years of age, and no invalid results. The 3 individuals under 20 years who had positive rapid test results may be positive due to exposure outside their community, or from residual transmission occurring over 10 years ago, however the possibility that these are false positives cannot be ruled out. While early prototypes of the Ov16 rapid test demonstrated a 97–98% specificity[18,24], the product insert states the performance of the commercially available Ov16 rapid test compared to skin snip microscopy in a laboratory setting to be 81.1% (95% CI: 70.7–88.4%) sensitive and 99.0% (95% CI: 94.8–99.8%) specific using whole blood, or 85.3% (95% CI: 75.6–91.6%) sensitive and 99.0% (95% CI: 94.7–99.8%) specific using serum and plasma (http://www.standardia.com/en/home/product/Rapid_Diagnostic_Test/Anti-Onchocerciasis_IgG4.html). Evaluation of the performance of the tool in the field is ongoing. Currently more data evaluating the sensitivity and specificity of the Ov16 rapid test to skin snip microscopy and ELISA in field settings is needed.

The difference in participation rates for Ov16 rapid test and skin snip microscopy suggests a greater willingness in these communities to undergo a rapid test with a finger prick compared to a more invasive skin snip procedure (99.7% and 32.7% respectively, p<0.0001). Some individuals who initially refused the skin snip biopsy later changed their mind and had the skin snip biopsy performed after learning their rapid test was positive. This may have resulted in an increased participation rate for skin snip than would have been seen otherwise, though the difference was likely small as there were relatively few positives. The 2016 WHO guidelines call out a need to further investigate the acceptability of skin snip microscopy in low prevalence settings. These findings align with others that observed high refusal rates for skin snip microscopy in similar settings.[9,13] The refusal of the skin snip biopsy in our study was largely due to not liking the idea of the test and considering the test to be too painful. Moreover, some community members suggested that they are less willing to undergo the biopsy if the results are consistently negative. The value of the different diagnostic tests during the distinct phases of elimination is important, and community members and rapid test technicians noted that skin snip microscopy remains the primary method for assessing infection status and recommending treatment, while the rapid test is a screening tool to inform decisions regarding the continuation or culmination of MDA.

Community members reported high levels of acceptability and willingness to participate in surveillance activities that included the rapid test. The role of the surveillance team and the information they provided influenced community members’ experience with the test. Clear communication about the test purpose, procedure, and result was appreciated by community members and increased their trust in the result and motivation to participate in other onchocerciasis control activities. The influence of the surveillance team should not be overlooked as they may be a valuable tool to encourage participation in future surveillance activities and greater compliance with mass drug administration. Community member feedback also showed that in areas endemic for onchocerciases and where consistent access to quality health services may be lacking, there is an appreciation for the delivery of health services through NTD control programs. Community members expressed a desire for greater knowledge of the health of their community, potential risk factors, and their achieved progress towards program goals.

The Ov16 rapid test is intended for use in populations nearing elimination. In this setting, the population is predominately healthy and unaffected by onchocerciasis. Attributes such as invasiveness of the test may be more important in these settings, particularly when testing is focused on children.

The costing analysis showed that the cost per person tested in this activity was $16.57 for the rapid test, $21.01 for the skin snip microscopy, and $24.15 for both methods. If no participants had refused the skin snip microscopy, the costs per participant using either method would have been comparable at around $16. The labor and instrument costs for skin snip microscopy were largely fixed and independent of the number of participants tested. The skin snip microscopy team had to remain with the study team for the duration of the surveillance activity regardless of how many people they were testing. Multiple skin snip microscopy instruments were used for this activity due to the need to sterilize equipment after each use, and these instruments were assumed to not be shared with other programs. Additionally, the rapid test had slightly lower training costs due to shorter training, but higher costs for devices and instruments that were dependent on the number of people tested. Performing skin snip microscopy on a subset of participants who are all receiving rapid testing is costly, due to the high costs of instruments and the need for two teams of technicians (rapid test technicians and skin snip technicians). This costing information may be useful when considering how to transition programs from skin snip to rapid testing. However, while comparable conclusions may arise from similar studies, the cost estimates from this study are specific to the Senegalese context. For example, costing results would vary based on country salary and per diem policies, the surveillance activity approach used such as number of days spent in the field and number of people tested per village. Similarly, different assumptions regarding the useful life of instruments for skin snipping would also affect the results. Additional implementation research is important in other locations to evaluate how results vary by setting.

A comprehensive quality assurance (QA) program including training videos and materials, quality assurance panels and daily quality control standards, was implemented along with the Ov16 rapid test to facilitate proper use of the test in this study. The QA program resources, which are freely available to implementing programs, minimize improper handling of the tests and user errors, while ensuring consistent product quality. Findings from this study also suggest this QA program may provide surveillance teams with greater confidence in technician skills and validity of the results, which benefits the community members by supporting the surveillance team in their dissemination of information and results. More research is needed to understand the role QA practices have on influencing user and participant confidence, identify QA best practices, and drive adoption of these practices through integration into global guidelines.[25]

**Fig 4:**
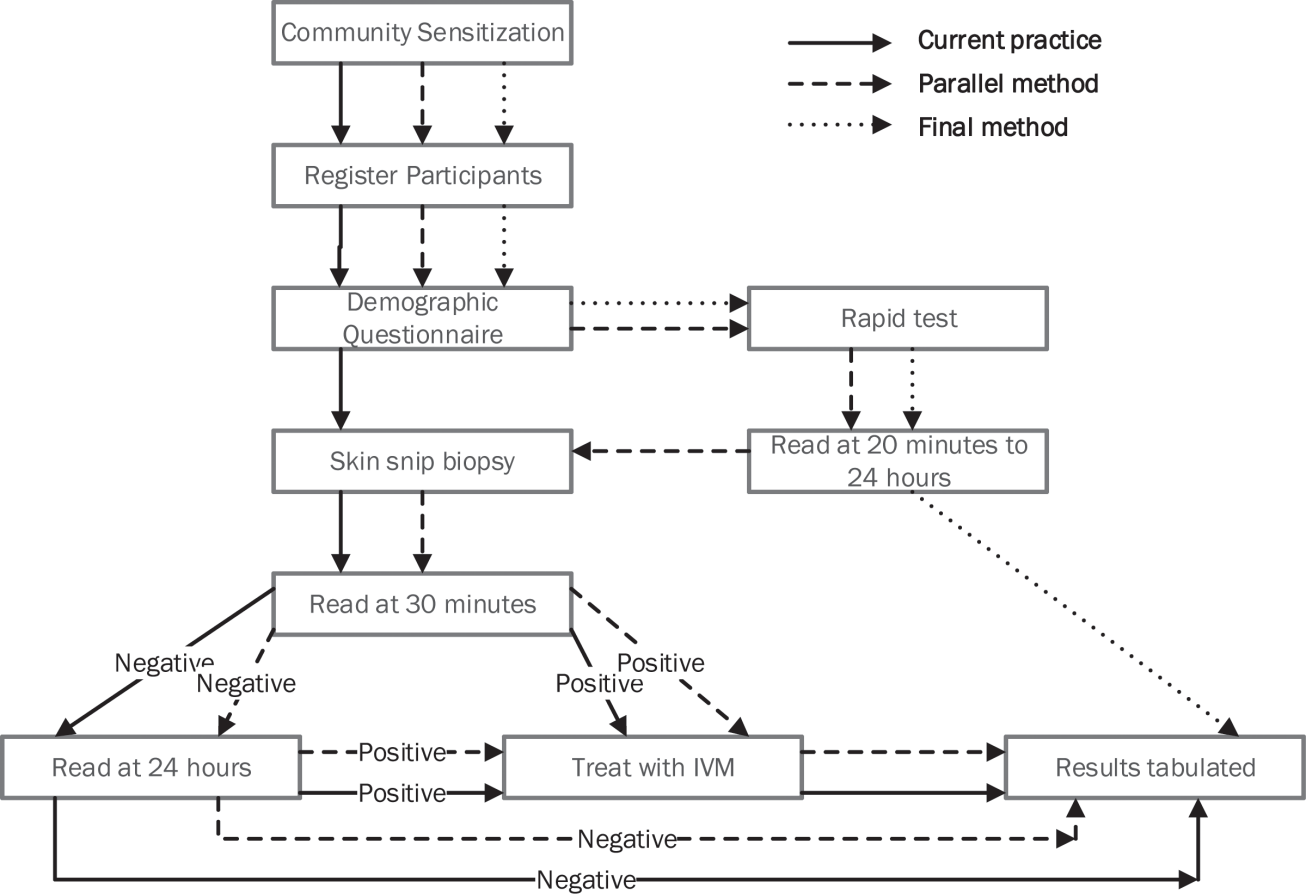
Onchocerciasis surveillance process map without and with Ov16 rapid test.

A process map illustrating the use of one or both tools in surveillance activities was generated from the 2015 workshop, this study, and the 2016 WHO guidelines. (Fig 4) The “current practice” uses skin snip microscopy only, a more relevant strategy in higher prevalence areas. The “parallel method” uses skin snip microscopy and Ov16 rapid test, which may be more appropriate when programs are transitioning to stopping MDA and implementing Ov16 serology such as in this study. However, in these transition areas, high refusal rates for skin snip may prevent programs from attaining a sufficient sample size to determine with certainty if program goals have been reached.[13] An alternative to the parallel method may be testing only Ov16 rapid test positives with skin snip microscopy, though in low prevalence settings few if any skin snip positives would likely be detected. The “final method” uses the rapid test only and “should be used in children under 10 years to demonstrate interruption of transmission”.[13] This study was not designed with a sampling methodology sufficient to determine prevalence or if transmission had been broken. According to guidelines, roughly 2000 children under 10 years of age would need to be tested to detect a prevalence of less than 0.1% with sufficient confidence, and only 368 children under 10 years were included in this study, all of whom were rapid test negative.[13]

Finally, diagnostic tools play an important role in influencing health outcomes, usually through the intended benefits of enabling timely diagnosis, accurate disease surveillance, and proper treatment. These tools may also have the ability to influence individual and community behaviors, such as participation in surveillance activities and confidence in control program activities. Taking a broader perspective, the true value of diagnostic tools may go beyond the intended utility to include extended benefits such as increased utilization of health care services, individual agency over the health care experience, and confidence in provider abilities. These broader benefits should be identified and measured in future implementation research to better understand how to move technologies beyond innovation and validation, and into adoption and scale-up.[19] Ov16 as a biomarker has successfully moved from discovery and development at the bench, to evidence of effectiveness in the field. The remaining barriers are optimized and context-specific integration into systems and programs. As global focus shifts to the integration of onchocerciasis and lymphatic filariasis (LF) programs to reach elimination in Africa faster, a rapid assessment of Ov transmission through LF transmission assessment surveys (TAS) will be required.[26] A more appropriate tool for this work may be the SD BIOLINE Oncho/LF IgG4 biplex rapid test to detect ongoing onchocerciasis and LF transmission simultaneously (http://www.standardia.com/en/home/product/Rapid_Diagnostic_Test/Oncho-LF_IgG4_biplex.html). [24] As more settings achieve success in control of either disease, integrated surveillance for transmission interruption may be a best-buy, and implementation research to support successful adoption and scale up is essential.

In this area of Senegal with low onchocerciasis sero-positivity, there was high participation with the rapid test, while participation with skin snip microscopy was significantly lower. Acceptability and perceived value of the rapid test was high among community members and rapid test technicians. The role of the surveillance team and the information they provided influenced community members’ trust in the result and motivation to participate. This may be a valuable tool to encourage participation in future surveillance activities and greater compliance with mass drug administration. This study provides evidence of the feasibility of implementing the Ov16 rapid test and the associate costs, which may be informative to other country programs interested in adopting this new tool as they move from control to elimination of onchocerciasis.

## Supporting information

S1 File Costing analysis. (PDF)

S2 File Quantitative data. (XLS)

S3 File STROBE checklist. (PDF)
