# Supplementary material for: Feasibility of utilizing the SD BIOLINE Onchocerciasis IgG4 rapid test in onchocerciasis surveillance in Senegal
Source: PLoS Negl Trop Dis. 2017 Oct 3;11(10):e0005884. doi: 10.1371/journal.pntd.0005884 (PMC5640270; doi:10.1371/journal.pntd.0005884)
Supplement: S1 File — (PDF) [file pntd.0005884.s001.pdf]

Supporting Information

Costing analysis

Study duration in the field: Field activities lasted 10 days, May 23 to June 1 2016, during which 13 villages were visited. The original timeline for field activities was 13 days, but due to upcoming rains, the team visited two villages per day on 3 days. This may have reduced the daily allowances costs proportionally.

Table 1. Field activities schedule and number of people tested per day

Village	Number of participants	% to total number of participants	# of RDT positives	# of RDT negatives	# who rejected RDT	# accepted skin snips	# rejected skin snips
Badon	46	4%	0	46	0	1	45
Bamboya	68	6%	1	67	0	1	67
Soucouta	108	9%	2	105	1	3	105
Mandathiès	100	9%	2	98	0	1	99
Yamoussa (Thiabekani)	46	4%	0	46	0	1	45
Thiancoumalal	91	8%	3	87	1	21	70
Badian	30	3%	0	30	0	3	27
Kabateguenda	84	7%	0	84	0	5	79
Ndjendi Bassiri	53	5%	4	49	0	27	26
Diakha Madina	142	12%	0	142	0	129	13
Bambadji	114	10%	3	111	0	88	26
Bofeto	78	7%	3	74	1	42	36
Missirah Dantila	152	13%	3	148	1	60	92
Total	**1,173**						

Training costs: Test-specific training included a half day for the rapid test team, and one full day for skin snip microscopy. In addition, the entire team participated in a half day training to organize the logistics of the surveillance activities. These costs included refreshments, venue rental, training material, per diems, and transport costs.

Field work costs: These costs included labor costs, transport costs, medical and non-medical supplies such as sterilizing reagents, instruments, and devices. To calculate labor costs, we multiplied local salaries by the number of days the staff participated in the surveillance activity, and also included labor costs associated with implementing the quality assurance program designed to support the rapid test (Table 2). The total estimated time for the quality assurance program was 15 hours, including 2 hours to perform the training panel, 2 hours for quality assurance panel, 1 hour for proficiency panel and 30 minutes a day for daily quality control). In addition to the per diem given, community health workers' time was valued using the annual agriculture value added per worker inflated to year 2016 (World Bank Development Indicators, World Bank), as previously done in a similar study.[27] Two vehicles were used for travelling to the villages: a PATH owned vehicle and a rented vehicle. Transport costs included total

actual expenses for the rented vehicle as well as fuel costs and vehicle depreciation costs for the days when PATH's vehicle was used (assuming linear depreciation over a 10-year lifetime). Transport costs also include per diems given to staff that covered lodging and daily allowance. All medical and non-medical supplies were identified and costs calculated by multiplying the number of units with the unit price. We also included the costs of 280 rapid test units to conduct the quality assurance program. Local unit prices were used for all items that could be purchased in Senegal, unless the item could only be purchased in the USA. This was the case for the skin snip tools which the Senegalese MOH requested. The annualized shipping costs per skin snip tool were $0.63 and were added to the analysis. Similarly, shipping costs for the rapid tests were $0.33 per test and were included in the analysis. All customers procuring the tests from the manufacturer will incur shipping costs, though the actual costs vary by location. This study did not include import duty, customs fees, or in-country logistics costs. Import duty and custom fees were waived for the present study, as it is often the case when a product is for government sponsored public health use. However, in some countries these costs may represent a significant percentage of the unit costs of the product. Instruments and medical devices that can be reused were assigned a useful life of ten (microscope and skin snip tools) or five years (glass slides, well-plates, etc.) based on inputs from the onchocerciasis team in Senegal. We assumed that these instruments and devices were only used for onchocerciasis surveillance activities, which happens once a year in Senegal.

Table 2: Labor costs

Job title	Number of staff with same job title	Number of days staff was involved	Daily allowance ($USD)*	Team
Laboratory technician	2	11	$25	RDT
EPI technician	2	11	$66	Skin snip
EPI technician	1	11	$66	Shared - consenting
ODK staff / Census officers[#]	2	11	$66	Shared – data entry
MoH team	2	12		Shared – general support
Community Health Worker	2	11	$9	Shared – surveillance
Nurse	1	1	$13	Shared - sensitization
Focal point	1	3	$25	Shared - sensitization
Drivers	3	11	$9	

*Daily allowance included per diems and lodging costs and we did not have enough details to separate the two components. The allowance for EPI technicians was significantly higher than the daily allowance for laboratory technicians due to the location of provenience. In this study, the higher lodging and transport costs partially offset the cost savings from lower skilled staff for RDT compared to EPI technicians.

[#]In the research study, ODK staff was in charge of collecting data in electronic form. For the costing study, we assume that the same staff would conduct the census outside the research context. It is possible that ODK staff is on average more educated than staff conducting census and this may inflate labor costs in the current analysis.

Data entry and reporting costs: These costs reflect costs for printing of paper documentation and labor costs for data processing such as entering and transmitting the collected data. Since the phone-based data collection that was used during this surveillance activity is not the standard practice in Senegal, we estimated costs under a scenario of paper-based data collection for all participants, with manual data

entry and computer-based data processing, based on the team's experience.

Costs of the instruments: The skin snip team used two microscopes for the detection of Ov microfilaria in the field and 15 skin snip tools. For instruments used in the Onchocerciasis program, we asked the focal person for Onchocerciases surveillance at the MoH to indicate which model the government would buy if they were to run the Onchocerciasis surveillance independently and asked them to provide the corresponding price. Since instruments can be used over multiple years, we assumed a useful life of 10 years and allocated only the costs of one year /one Onchocerciasis surveillance activity to this analysis. For other items lasting more than one that were also used in the field, we assume a useful life of 5 years (e.g. glass slides, 96—well-plates, cooking pots, etc.). The same approach was used to allocate the costs. We did not have information on whether the instruments were used for other programs as well; therefore, all costs were allocated to the Onchocerciasis surveillance program.

Table 3. Detailed unit prices for costing analysis and data sources by testing method.

Item	Unit price ($US), 2016	Quantity	Data source
**Ov16 rapid test**			
Ov16 rapid test	$1.20	1,690 (includes tests for QA)	Manufacturer website: http://www.standardia.com/en/home/product/Rapid_Diagnostic_Test/Anti-Onchocerciasis_IgG4.html
Ov16 rapid test shipping cost to Dakar, Senegal	$ 0.33	na	Based on Standard Diagnostics, Inc. (manufacturer) shipping bill paid by PATH for this study.
VWR absorbent undepads	$19	5	Study expense reports / receipts
Safety boxes	$4.50	24	Study expense reports / receipts
D.O.T. labels "Biohazard"	$29	1 roll	Study expense reports / receipts
**Skin snip test**			
Microscope	$2,490	2	Based on input from Senegalese onchocerciasis team regarding type of microscope needed
Skin snip tool	$225	15	Based on cost of tools procured for this study. Manufacturer website: http://www.medicaldevicepurchase.com/MP-811-Holth-Corneoscleral-Punch-Prodview.html
Skin snip tool (annualized) shipping cost to Dakar, Senegal	$ 0.63	na	Based on manufacturer website: http://www.medicaldevicepurchase.com/MP-811-Holth-Corneoscleral-Punch-Prodview.html
Glass slides	$3.55	75	Study expense reports / receipts
Lamelle	$0.50	100	Study expense reports / receipts
96-well plates	$2.20	4	Study expense reports / receipts
Sterilizing pot	$5	1	Study expense reports / receipts
Gas burner	$6.80	4	Study expense reports / receipts
Alcohol	$6.80	4 bottles, 500ml	Study expense reports / receipts
Compresses	$0.03	3500 pieces	Study expense reports / receipts
Syringes	$8.45	1 box of 100 units	Study expense reports / receipts
Cleaning solvent	$2.20	2 bottles	Study expense reports / receipts
Disinfectant	$0.80	4 bottles	Study expense reports / receipts

Distilled water	$5	NA	Study expense reports / receipts
Butane	$6.80	6 bottles	Study expense reports / receipts
**Office supplies**			
Miscellaneous	$285		Study expense reports / receipts
